# Emerging Role of Ferroptosis in Diabetes and Associated Complications: When Metabolic Dysregulation Meets Cell Death

**DOI:** 10.1111/cpr.70187

**Published:** 2026-02-25

**Authors:** Zheng Wang, Ying Zhang, Yue Xu, Mengyuan Yang, Yuan Wei, Yuan Li, Tongyue Yang, Xu Wang, Chaona Yang, Qi Feng, Guolan Xing

**Affiliations:** ^1^ Department of Nephrology The First Affiliated Hospital of Zhengzhou University Zhengzhou People's Republic of China; ^2^ Academy of Medical Sciences Zhengzhou University Zhengzhou People's Republic of China; ^3^ Henan Province Research Center for Kidney Disease The First Affiliated Hospital of Zhengzhou University Zhengzhou People's Republic of China; ^4^ Division of Endocrinology, Department of Internal Medicine The First Affiliated Hospital of Zhengzhou University Zhengzhou China; ^5^ Clinical Medical College of Henan University of Science and Technology Luoyang People's Republic of China

**Keywords:** diabetes mellitus (DM), diabetic complications, ferroptosis, molecular mechanisms, research progress

## Abstract

Diabetes mellitus (DM) is a metabolic disorder marked by persistent hyperglycemia (HG), resulting from abnormalities in insulin secretion or insulin resistance. This condition represents a major public health concern since it causes multisystem complications, including microvascular diseases, macrovascular diseases, and neuropathy. Few effective therapies are currently available. Ferroptosis, an iron‐dependent mode of regulated cell death triggered by lipid peroxidation (LPO), is intricately linked to the pathogenesis, progression, and complications of DM. It has been increasingly recognised as a key mechanism underlying peripheral insulin resistance and insulin deficiency resulting from β‐cell dysfunction. In this study, we systematically summarised the primary regulatory mechanisms of ferroptosis and outlined current research advancements in mechanistic insights into its role in diabetic complications. Besides, we explored how inter‐organelle interactions drive ferroptosis under diabetic conditions and play pathogenic effects in diabetes and its complications. Finally, we systematically reviewed the therapeutic drugs targeting ferroptosis from the perspectives of traditional Chinese medicine (TCM) and Western medicine, respectively. This interdisciplinary integrated overview may provide a theoretical basis for future clinical transformation.

## Graphical abstract

This study identifies ferroptosis as a key driver of diabetes and its complications via iron metabolism and lipid peroxidation, elucidates organelle interactions underlying cell vulnerability, and provides insights for targeted therapies against metabolic disorders.

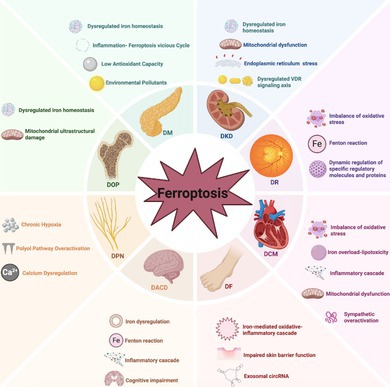

## 1 Introduction

Globally, diabetes mellitus (DM) is among the chronic diseases exhibiting the fastest growth rate. The International Diabetes Federation (IDF) forecasts an increase in the global diabetic population from 537 million in 2021 to 643 million by 2030, and exceeding 783 million by 2045 [1], imposing a major burden on socioeconomic systems and individual health. Based on its etiopathogenesis, diabetes can be categorised into two major types: type 1 diabetes (T1DM), which is characterised by islet β‐cell death and complete insulin deficiency, and type 2 diabetes (T2DM), dominated by insulin resistance and progressive insulin secretion dysfunction [2]. Although glycemic control has greatly improved due to advances in diabetes research and ongoing clinical guidelines, most patients still face inevitable progression to macrovascular disorders like cardiovascular diseases and microvascular diseases like diabetic kidney disease (DKD) and retinopathy [3]. These issues frequently result in decreased quality of life, organ failure, and even death. Accordingly, a better understanding of the inherent pathophysiology as well as the development of targeted therapies are crucial.

The three main characteristics of ferroptosis‐iron overload, increased lipid peroxidation (LPO), and suppression of glutathione peroxidase 4 (GPX4) activity‐were initially identified in 2012 [4]. Morphologically, ferroptosis is characterised by increased mitochondrial membrane density, decreased mitochondrial cristae, and mitochondrial atrophy. Iron‐dependent LPO and the accumulation of reactive oxygen species (ROS) represent hallmarks of ferroptosis [5]. Current evidence suggests that ferroptosis pathogenesis is involves dysregulated iron homeostasis, lipid metabolism, and antioxidant defence, contributing to multifaceted pathologies such as cancer, neurological disorders, and ischemia–reperfusion injuries [6]. Research on the impact of ferroptosis in diabetes and its consequences has gained significant momentum, given that new evidence suggests that DM is often linked to iron metabolism abnormalities [7]. Elucidating the regulatory mechanisms of ferroptosis in diabetes and its complications presents a promising pathway for developing novel treatments to improve clinical outcomes and comorbidity management.

In this review, we update the contribution of ferroptosis in the pathogenesis of diabetes and its complications, elucidating for the first time how organelle interactions drive the progression of ferroptosis under diabetes and exploring its pathogenic effects. We additionally present the inaugural comprehensive overview of therapeutic strategies targeting ferroptosis from both western and TCM perspectives, which may serve as a foundational basis for preventive clinical applications.

## 2 Molecular Mechanisms of Ferroptosis

**FIGURE 1:**
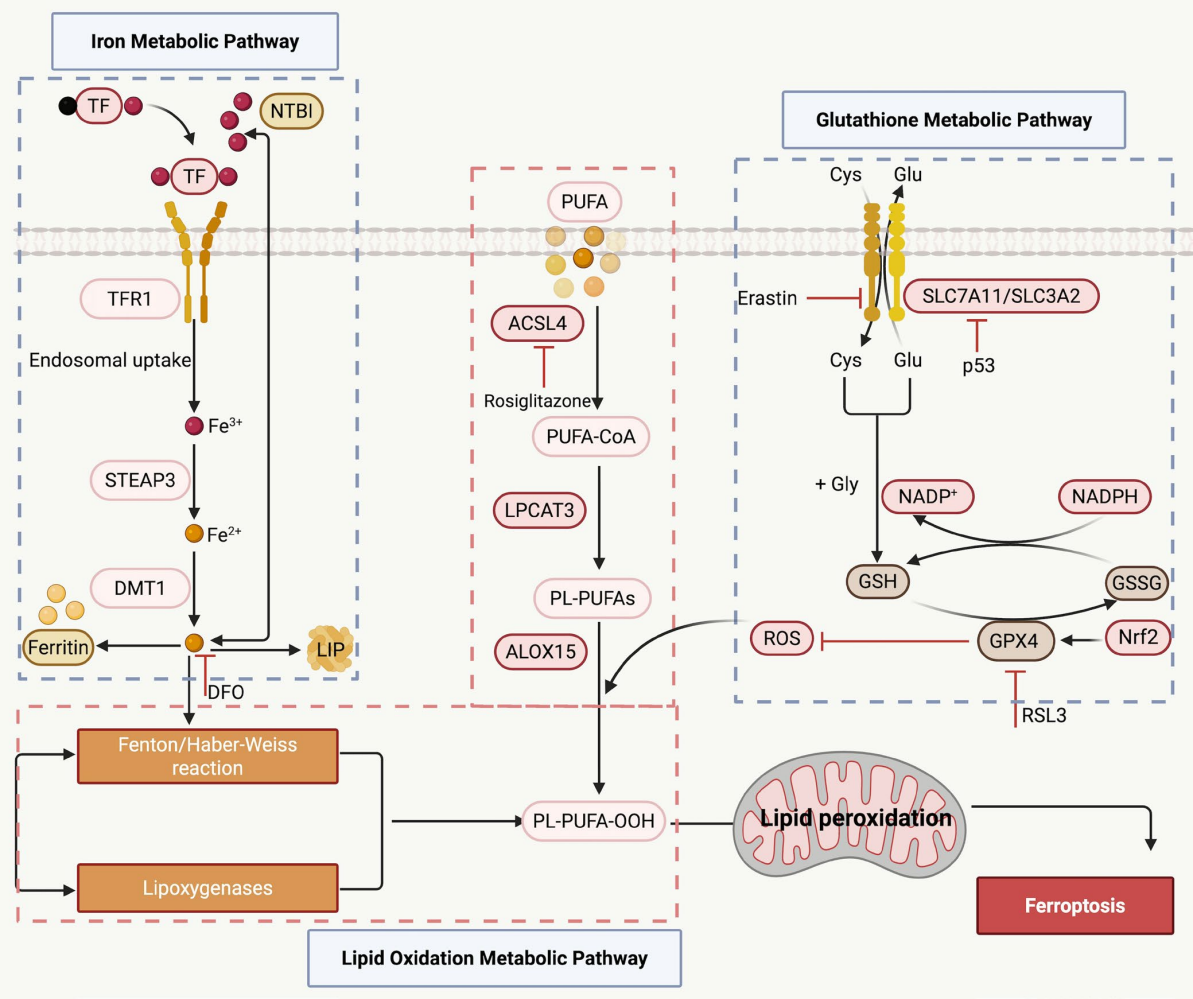
Core signalling pathways and regulators of ferroptosis in DM and its complications. Ferroptosis is induced by the accumulation of PUFA‐PL peroxides, with dysregulation of iron metabolism and LPO and inactivation of antioxidant defence systems, collectively constituting the core molecular mechanisms underlying ferroptosis occurrence. ACSL4, acyl‐CoA synthetase long‐chain family member 4; ALOX15, arachidonate lipoxygenase 15; DFO, deferoxamine; DMT1, divalent metal transporter 1; GPX4, glutathione peroxidase 4; GSH, glutathione; GSSG, glutathione disulphide; LIP, labile iron pool; LPCAT3, lysophosphatidylcholine acyltransferase 3; NADP^+^, nicotinamide adenine dinucleotide phosphate; NADPH, reduced nicotinamide adenine dinucleotide phosphate; Nrf2, nuclear factor erythroid‐2‐related factor 2; NTBI, nontransferrin‐bound iron; PL‐PUFA‐OOH, phospholipid‐bound polyunsaturated fatty acid hydroperoxides; PUFA‐PL, polyunsaturated fatty acid‐containing phospholipid; PUFAs, polyunsaturated fatty acids; RSL3, Ras‐selective lethal 3; SLC3A2, solute carrier family 3 member 2; SLC7A11, solute carrier family 7 member 11; STEAP3, six‐transmembrane epithelial antigen of prostate 3; TF, transferrin; TFR1, transferrin receptor 1.

Ferroptosis differs from other forms of regulated cell death, such as apoptosis and autophagy, due to specific physical and chemical characteristics. It is controlled by the interplay of metabolic pathways, including dysregulation of lipid metabolism, aberrant iron metabolism, and imbalance in the glutathione (GSH) antioxidant system. In the following section, we provide a brief overview of the primary mechanisms that cause ferroptosis in DM and their effects (Figure 1).

### 2.1 Iron Metabolism Dysregulation

In the human body, iron is an essential trace element vital for physiological functions such as oxygen transport and DNA synthesis [8]. Circulating Fe^3+^ forms a transferrin (TF)‐Fe^3+^ complex with TF on the cell membrane under normal physiological conditions. This complex is then internalised into cells through receptor‐mediated endocytosis via transferrin receptor 1 (TFR1). In the cell nucleus, prostate six‐transmembrane epithelial antigen of prostate 3 (STEAP3) of the cell converts Fe^3+^ to Fe^2+^. The divalent metal transporter 1 (DMT1) then transports Fe^2+^ into ferritin and the cytoplasmic labile iron pool (LIP) [9]. To avoid iron overload, ferroprotein 1 (FPN1) oxidises excess Fe^2+^ to Fe^3+^ and exports it from the cell [10]. Current evidence suggests that cellular damage results from saturated TF's promotion of nontransferrin‐bound iron (NTBI) inflow through DMT1 during iron overload [11, 12]. Furthermore, excessive Fe^2+^ triggers Fenton and Haber‐Weiss reactions, which generate lipid peroxides and cause ferroptosis [6, 13]. Diseases such as DM [14], liver fibrosis [15], cardiomyopathy [16], heart failure [17], neurological disorders [18], and cancer [19] are all closely linked to iron overload. Damage from ferroptosis can be mitigated by iron chelators such as deferoxamine (DFO) [20]. Furthermore, reducing NTBI absorption by targeting DMT1 is a viable treatment approach. Overall, a comprehensive understanding of iron homeostasis is crucial for identifying the underlying causes of ferroptosis and developing effective treatment strategies for DM and its complications.

### 2.2 LPO

Iron‐dependent LPO builds up due to oxidative degradation of polyunsaturated fatty acids (PUFAs). The susceptibility of PUFAs to oxidation, a consequence of their unstable double bonds, leads to the formation of lipid hydroperoxides (PUFA‐OOH). This process damages cellular membranes and promotes ferroptosis. Abnormal lipid metabolism is closely associated with ferroptosis [21, 22]. This process involves two key enzymes: activated acyl‐CoA synthetase long‐chain family member 4 (ACSL4), which facilitates the esterification of PUFAs to form PUFA‐CoA, and lysophosphatidylcholine acyltransferase 3 (LPCAT3), which aids in integrating these esterified PUFAs into membrane phospholipids. When lipoxygenases oxidise these lipid molecules, especially arachidonate lipoxygenase 15 (ALOX15), LPO is produced. Eventually, excessive accumulation of LPO impairs membrane integrity and causes ferroptosis [23, 24]. Moreover, iron‐catalysed non‐enzymatic LPO constitutes a significant source of LPO [25]. Ferroptosis is ultimately triggered when the intracellular accumulation of lipid peroxides surpasses the cell's intrinsic antioxidant clearance capacity. One important strategy for controlling ferroptosis is to downregulate LPCAT3 expression or inhibit ACSL4 function. Conversely, increased LPCAT3 and ACSL4 expression or activity may promote ferroptosis. Collectively, these findings shed light on the crucial role of LPO in the molecular mechanisms of ferroptosis and propose potential therapeutic targets.

### 2.3 Dysregulation of the Glutathione Antioxidant System

Current evidence suggests that ferroptosis arises from imbalances between cellular antioxidant defences and oxidative stress [26]. Cysteine uptake and reduction to cysteine, a crucial precursor for GSH synthesis, are mediated by the cystine/glutamate antiporter System Xc‐, a cystine/glutamate blocking system [4], which preserves GSH homeostasis, a key precursor for GSH synthesis, thereby maintaining GSH homeostasis [27]. GSH acts as an essential cofactor for GPX4, catalysing the conversion of phospholipid hydroperoxides (PLOOH) to nontoxic phospholipid alcohols (PL‐OH), thereby neutralising lipid peroxides [28]. This process converts GSH into oxidised glutathione (GSSG), which is subsequently replenished by GSH disulphide reductase to preserve the antioxidant cycle [29, 30]. When this pathway is disrupted, whether through GSH depletion or inhibition of GPX4 activity by Ras‐selective lethal 3 (RSL3), LPO products accumulate and ultimately result in ferroptosis [31, 32]. Ferroptosis can be suppressed by the transsulfuration pathway, which is mediated by the System Xc‐ [33] and glutaminolysis [34], which restores GSH and strengthens antioxidant capacity. Overall, the system Xc‐/GSH/GPX4 axis is one of the primary regulatory pathways in ferroptosis. Its dysfunction, specifically involving GPX4 depletion or enzymatic inactivation, is directly linked to the onset of ferroptosis, emphasising its promise as a therapeutic target. Interestingly, the observation that ferroptosis can be triggered independently by depleting cytosolic or mitochondrial GSH underscores the complexity of its regulatory network.

### 2.4 Other Regulatory Mechanisms

Research has shown that ferroptosis cannot be induced by GPX4 deletion alone [35], suggesting the presence of compensatory mechanisms independent of GPX4. During acute kidney injury (AKI), the ferroptosis suppressor protein 1 (FSP1)‐coenzyme Q10 (CoQ10)‐nicotinamide adenine dinucleotide phosphate (NADPH) pathway prevents ferroptosis in tubular epithelial cells, which yields protective effects [36]. Similarly, in amyotrophic lateral sclerosis (ALS), the GTP cyclohydrolase 1 (GCH1)/tetrahydrobiopterin (BH4) axis prevents neuronal ferroptosis by reducing LPO [37]. Interestingly, a study revealed that dihydroorotate dehydrogenase (DHODH) inhibits LPO by regulating mitochondrial CoQ10 levels [38, 39]. Moreover, membrane‐bound O‐acyltransferase 1/2 (MBOAT1/2) maintains membrane lipid homeostasis through phospholipid remodelling that is dependent on sex hormone receptors [40]. The existence of multiple compensatory pathways suggests that synergistic targeting of key nodes, such as GPX4, FSP1, and GCH1, may increase the efficacy of ferroptosis‐based treatment approaches, even though the FSP1/CoQ10 and Xc‐/GSH/GPX4 systems are the primary defence mechanisms [37].

Ferroptosis is regulated by signalling pathways like adenosine monophosphate‐activated protein kinase (AMPK), nuclear factor erythroid‐2‐related factor 2 (Nrf2), and tumour protein 53 (p53). Among these, p53 exhibits bidirectional regulation characteristics [41, 42], indicating its functional dependence on the cellular environment. Important regulators of mitochondrial metabolism, such as voltage‐dependent anion channels (VDACs), can significantly reduce intracellular ROS and LPO product levels by blocking VDAC oligomerization, which in turn reduces hepatocyte ferroptosis [43].

Therefore, the core mechanism of ferroptosis lies in iron homeostasis imbalance and ROS‐dependent LPO accumulation. A key strategy to inhibit ferroptosis involves protecting the cell's capacity to resist LPO, particularly the function of GPX4.

## 3 Cross‐Regulation Between Organelle Interactions and Ferroptosis

**FIGURE 2:**
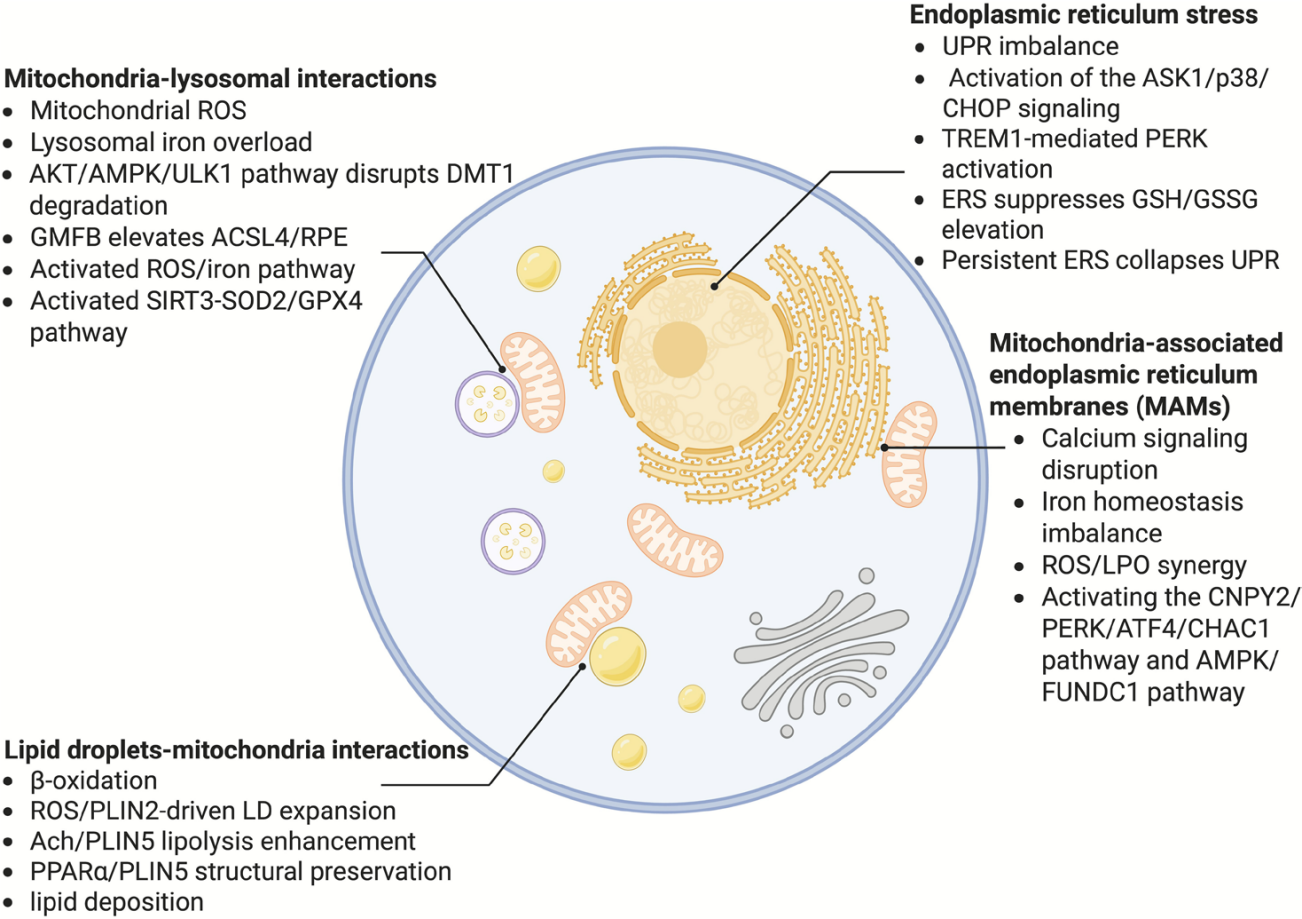
Cross‐regulation between organelle interactions and ferroptosis. The malfunction of several organelles, including ERS, MAMs, MLIs, and LD‐MIs, affects the susceptibility of cells to ferroptosis. Ach/PLIN5, acetylcholine/perilipin 5; AKT/AMPK/ULK1, protein kinase B/adenosine monophosphate‐activated protein kinase/UNC‐51‐like kinases 1; AMPK/FUNDCI, adenosine monophosphate‐activated protein kinase/FUN14 domain containing 1; ASK1/p38/CHOP, apoptosis signal‐regulating kinase 1/protein 38/C/EBP homologous protein; CNPY2/PERK/ATF4/CHAC1, canopy FGF signalling regulator 2/protein kinase RNA‐like endoplasmic reticulum kinase/activating transcription factor 4/glutathione‐specific gamma‐glutamylcyclotransferase 1; ERS, endoplasmic reticulum stress; GMFB, Glial maturation factor β; PPARα, peroxisome proliferator‐activated receptor α; ROS/PLIN2, Reactive oxygen species/perilipin 2; RPE, retinal pigment epithelium; SIRT3‐SOD2/GPX4, sirtuin‐3‐deacetylates Mn‐SOD/glutathione peroxidase 4; TREM1, triggering receptor expressed on myeloid cells 1; UPR, unfolded protein response.

In recent years, the role of interorganelle crosstalk mediated by proteins, RNAs, or metabolites in regulating ferroptosis and its pathological implications in diabetes and its complications has emerged as a major research focus. Herein, we briefly outline the primary mechanisms underlying this process (Figure 2).

### 3.1 Mitochondria‐Associated Endoplasmic Reticulum Membranes (MAMs) and Ferroptosis

MAMs are dynamic contact sites between mitochondria and the ER that are essential for iron homeostasis, lipid metabolism, signal transduction, and mitochondrial fusion/fission dynamics [28, 44, 45, 46]. Research on the regulation of ferroptosis and diabetes‐related disorders has focused heavily on the role of interorganelle crosstalk via proteins, RNAs, or metabolites.

Alterations in the function of MAMs are widely thought to result in diabetic cardiomyopathy (DCM) and DKD. Numerous dysfunctions, including ROS‐driven LPO, disrupted iron homeostasis, and aberrant calcium signalling, aggravate damage from diabetes. Overexpression of canopy FGF signalling regulator 2 (CNPY2) in renal tubules in DKD induces endoplasmic reticulum stress–related signalling, leading to MAMs dysfunction and renal tubular injury. CNPY2 overexpression worsens outcomes in *db/db* animals, whereas CNPY2 knockdown decreases renal damage, improves MAMs integrity, and decreases ferroptosis [47]. In the context of DCM, HG suppresses AMPK function while upregulating the expression of FUN14 domain containing 1 (FUNDC1) and inositol 1,4,5‐trisphosphate receptor type 2 (IP3R2). FUNDC1 stabilises IP3R2 by inhibiting ubiquitination, forming MAMs, and causing abnormal Ca^2+^ flow between the endoplasmic reticulum and mitochondria. This leads to structural fragmentation, ROS production, LPO, and mitochondrial Ca^2+^ overload [48]. Moreover, AMPK inactivation may potentiate damage caused by ferroptosis, as well as interfere with antioxidant pathways and iron homeostasis. MAMs integrity stabilisation via FUNDC1 exacerbates oxidative stress, while mitochondrial Ca^2+^ excess causes LPO. Accordingly, targeting MAMs may open new treatment options to halt the progression and complications of diabetes.

### 3.2 Endoplasmic Reticulum Stress (ERS) and Ferroptosis

It is now understood that ERS, which is caused by impaired endoplasmic reticulum function, is directly related to the onset and development of diabetes [49]. Interestingly, in diabetes, the interplay between ferroptosis and ERS plays an essential role in contributing to cellular damage. Excess iron affects glucose metabolism by initiating the apoptosis signal‐regulating kinase 1 (ASK1)/protein 38 (p38)/C/EBP homologous protein (CHOP) pathway, leading to ferroptosis in pancreatic β cells, as indicated by research [50]. One important transcriptional regulator of ERS, CHOP, plays a crucial role in this pathogenic process. According to another study on diabetes‐associated cognitive dysfunction (DACD) by Zhao et al., microglia under HG conditions activate the triggering receptor expressed on myeloid cells 1 (TREM1). Following this, TREM1 activates the ERS‐associated PERK pathway, which inhibits antioxidant defences and promotes aberrant intracellular iron accumulation, as evidenced by elevated GSSG and decreased GSH. This process amplifies LPO and induces microglial ferroptosis via increased ROS and malondialdehyde (MDA) [51]. Chronic ferroptosis exacerbates neuroinflammation and oxidative stress, ultimately leading to cognitive impairment.

Natural antioxidants such as resveratrol have demonstrated the ability to inhibit ferroptosis and preserve pancreatic β‐cell function by reducing ERS and enhancing the expression of peroxisome proliferator‐activated receptor gamma (PPARγ) [52]. Overall, these findings establish the theoretical basis for formulating treatment strategies that address the interaction between ferroptosis and ERS, yielding new insights into the pathophysiology of diabetes.

### 3.3 Mitochondria–Lysosomal Interactions (MLIs) and Ferroptosis

Mitochondrial dysfunction accelerates ferroptosis in diabetes by increasing oxidative stress and damaging cells [53]. Studies have demonstrated that the overexpression of Caveolin‐1 (Cav‐1) decreases DACD by modifying mitochondrial homeostasis associated with neuronal ferroptosis [54]. Besides, N‐acetylcysteine (NAC) prevents ferroptosis‐driven degenerative damage in diabetic nephropathy by triggering the Sirtuin‐3 deacetylates Mn‐SOD (SIRT3‐SOD2)/GPX4 pathway, thereby preserving mitochondrial redox homeostasis [55].

Abnormal iron accumulation within lysosomes, which function as iron storage depots, directly precipitates ferroptosis in diabetes by disrupting iron metabolism and promoting LPO [56]. In diabetic glucose deprivation, the protein kinase B (AKT)/AMPK/UNC‐51‐like kinases 1 (ULK1) pathway increases the autophagy proteins p62 and LC3, which inhibit the lysosomal membrane protein LAMP2 and promote the accumulation of the damage marker LGALS3. This leads to increased ferritin heavy chain 1 (FTH1) and system Xc, decreased lysosomal degradation of the iron transporter DMT1, and intralysosomal iron accumulation, all of which accelerate ferroptosis [57]. Glial maturation factor β (GMFB), which is released by Müller cells in diabetic retinopathy (DR) under HG conditions, disrupts lysosomal integrity and function, thereby facilitating ACSL4‐dependent lipid peroxide accumulation and retinal pigment epithelium (RPE) cell ferroptosis [58].

Current evidence suggests that in diabetes, lysosomes and mitochondria independently regulate ferroptosis and its associated effects. Dynamic organelle contact during ferroptosis has been demonstrated by optical molecular probes, despite the lack of direct evidence of crosstalk between mitochondria and lysosomes [59]. Although elevated mitochondrial ROS causes ferroptosis and destabilises lysosomal integrity, lysosomal dysfunction can exacerbate LPO by increasing mitochondrial ROS. This mutual control suggests that communication between mitochondria and lysosomes may play a role in diabetes disorders, providing a target for therapeutic strategies aimed at preventing ferroptosis.

### 3.4 Lipid Droplet–Mitochondrial Interactions (LD‐MIs) and Ferroptosis

It is well‐established that lipid droplets (LDs), lipid reservoirs, storage‐release balance, and interorganellar interactions influence ferroptosis susceptibility [60]. LDs physically interact with mitochondria via cristae to form perilipin (PLIN)‐associated mitochondrial domains [61]. This direct physical contact facilitates the transfer of fatty acids, which is necessary for mitochondrial β‐oxidation [62]. Mitochondrial ROS upregulates LD‐associated proteins, including PLIN2 which promotes lipid accumulation and alters the oxidative metabolic balance of lipids, ultimately leading to ferroptosis [63, 64].

In DCM animal models, acetylcholine has been found to improve LD‐MIs by upregulating PLIN5, which prevents palmitate from inducing apoptosis in newborn rat ventricular cardiomyocytes [65]. Disrupted LD‐mitochondrial connections have also been linked to renal lipid deposition in DKD. Sesamol has been found to reduce renal lipotoxicity by stimulating the peroxisome proliferator‐activated receptor α (PPARα)/PLIN5 pathway to maintain the structural integrity of LD‐MIs [66]. This study identified that LD‐MIs represent the primary therapeutic target for lowering ectopic lipid deposition in DKD.

Ferroptosis is widely understood to be associated with the onset, progression, and clinical outcomes of DM [14]. Although LD‐mitochondrial contacts may regulate lipid metabolic balance, their molecular role in ferroptosis requires experimental validation. Investigating the synergistic mechanisms of LD‐MIs, such as metabolic signalling, lipid dynamics, and ROS regulation, may help improve our understanding of ferroptosis‐driven diabetic pathologies. This knowledge would support therapeutic strategies aimed at mitigating metabolic dysfunction in LD‐MIs.

Currently, the role of organelle interactions in driving ferroptosis under diabetic conditions remains a hot topic requiring further investigation. However, existing research data suggest that targeting the regulatory mechanisms of organelle interactions and ferroptosis may offer novel therapeutic strategies for diabetes and its complications.

## 4 Ferroptosis in DM and Its Complications

**FIGURE 3:**
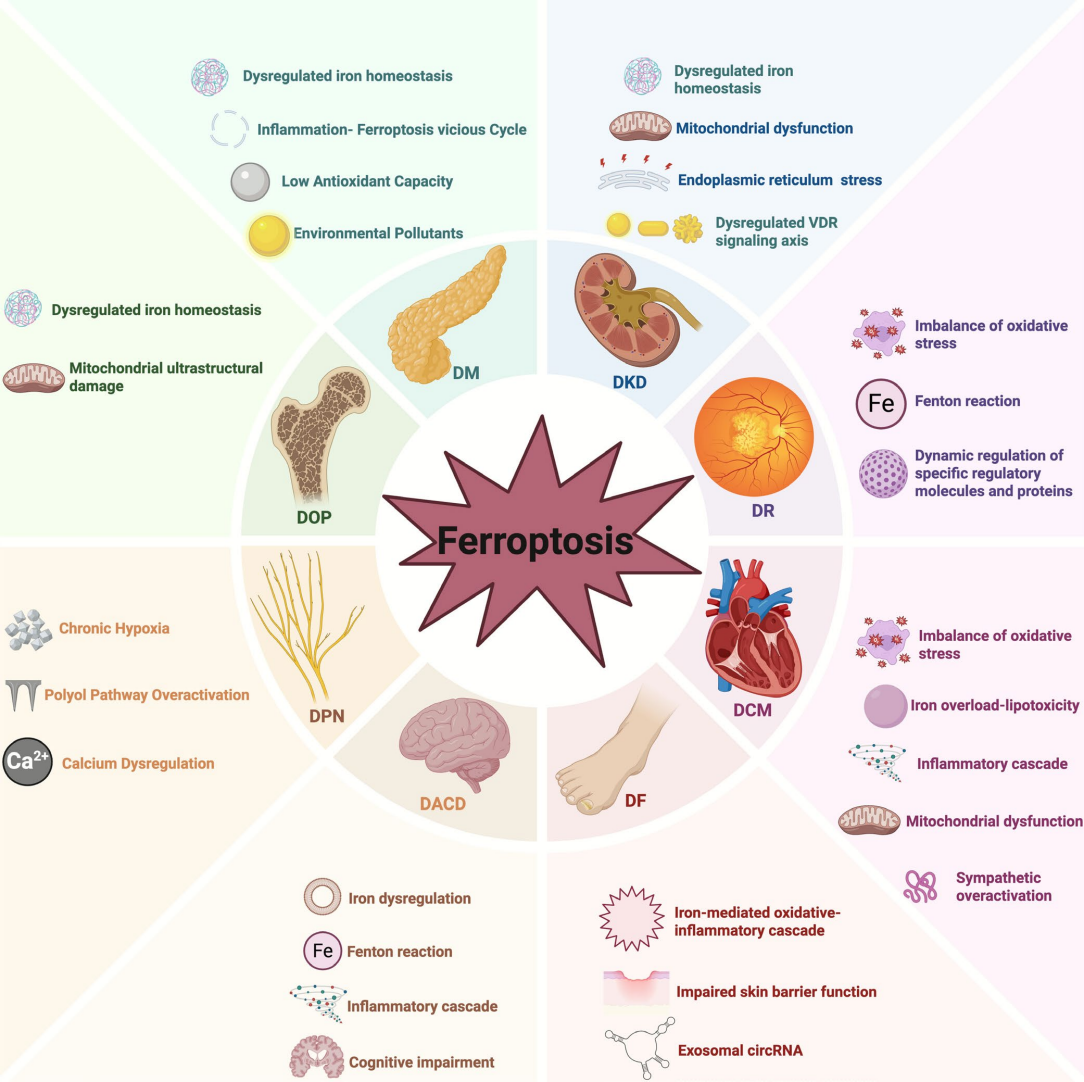
Ferroptosis in DM and its complications. The development of DM and its complications, such as diabetic retinopathy (DR), diabetic cardiomyopathy (DCM), diabetic foot (DF), diabetic peripheral neuropathy (DPN), and diabetic osteoporosis (DOP), is accelerated by ferroptosis‐mediated dysregulation of iron, lipid metabolism, and antioxidant defences in target cells.

Ferroptosis significantly contributes to the aetiology of various diseases. This section elaborates on the fundamental molecular mechanisms of ferroptosis in diabetes and its related effects. (Figure 3).

### 4.1 Ferroptosis and DM

Diabetes, a metabolic disease featuring insulin resistance and β‐cell dysfunction, is defined by chronic low‐grade inflammation [67]. Ferroptosis is mediated by inflammatory pathways activated by proinflammatory cytokines, such as Interleukin (IL)‐6, IL‐1β, and IL‐18. Conversely, HG exacerbates endothelial cell ferroptosis through the p53‐system Xc‐GSH axis, resulting in a vicious cycle [68, 69, 70]. Insulin secretion, insulin sensitivity, and metabolic homeostasis are all directly impacted by the positive feedback loop that iron metabolism produces through Fe‐S cluster production and management of free iron [71, 72, 73, 74, 75]. Additionally, a LIP resulting from iron metabolism imbalance can increase ROS production via the Fenton reaction and trigger LPO cascades by inhibiting GPX4 activity, ultimately leading to iron‐induced β‐cell injury [76]. Therefore, the functional integrity of β‐cell function depends on iron metabolism.

Individuals with T2DM often exhibit dysregulation of iron homeostasis. Iron overload directly impacts pancreatic β‐cell function through iron accumulation [76], which increases the risk of diabetes [77]. Ferroptosis, which is accelerated by β‐6 polyunsaturated fatty acid (PUFA)‐driven LPO, can be mitigated by the iron chelator DFO. The low glutathione peroxidase activity of β‐cells is approximately 15% of liver levels [78, 79]. Besides, acrolein directly causes GPX4 depletion and accumulation of LPO products; cadmium causes iron accumulation, GSH depletion, and mitochondrial damage through the GPX4/Ager/p65 axis, and arsenic disrupts pancreatic function through mitochondrial ROS‐mediated autophagy–lysosomal pathways [80, 81]. However, this cellular toxicity is potentially counteracted by natural compounds. For instance, resveratrol reverses this damage, while quercetin (QCT) may protect T2DM patients from β‐cell ferroptosis by preventing LPO, GPX4 inactivation, iron deposition, and GSH depletion [82]. These results collectively demonstrate the significant therapeutic potential of these chemical agents.

Current evidence suggests that iron dysregulation in β‐cells is a critical mechanism in the pathophysiology of T2DM. Accordingly, focusing on iron homeostasis to preserve β‐cell integrity and enhance insulin secretion and sensitivity can lead to novel approaches to diabetes treatment.

### 4.2 Ferroptosis and DKD

DKD is a severe microvascular complication of diabetes [83], typically characterised by proteinuria and a gradual decline in renal function, ultimately progressing to end‐stage renal disease [84]. The pathological changes in DKD represent a complex and progressive process. Early stages are primarily marked by glomerular hypertrophy and thickening of the basement membrane, while late stages are characterised by glomerulosclerosis, tubular atrophy, and interstitial fibrosis [83, 85, 86, 87, 88]. These alterations, under the sustained influence of diabetes, progressively impair renal function. Accumulating evidence suggests that inflammation induced by HG, dysfunction of the renin‐angiotensin system, and disruption of the glucose‐lipid metabolism‐oxidative stress axis play key roles in the pathogenesis [89, 90, 91].

Accumulating evidence highlights ferroptosis as a pivotal driver of tubular cell death under diabetic conditions. In diabetic models, renal expression of the system Xc‐ and GPX4 is markedly reduced, accompanied by decreased glutathione and elevated LPO; these alterations are effectively reversed by the ferroptosis inhibitor Ferrostatin‐1, which notably improves renal function and attenuates fibrosis in db/db mice [92, 93]. In parallel, ERS enhances HK‐2 cell sensitivity to ferroptosis via Nrf2 ubiquitination, promoting ROS accumulation, iron overload, and LPO, thereby driving epithelial‐mesenchymal transition and tubulointerstitial fibrosis in DKD [94]. Notably, dapagliflozin (DAPA) has been shown to protect against ferroptosis‐associated tubular mitochondrial membrane rupture and the cristae loss by stabilising SLC40A1, an effect independent of glycemia control [95].

Ferroptosis also exerts a critical impact in the glomerulus, particularly in early‐stage DKD, where Nrf2 upregulation enhances the expression of ferroptosis‐related targets [GPX4, FTH‐1, Solute carrier family 7 member 11 (SLC7A11)] and reduces TFR‐1 expression. These regulatory effects stabilise the cytoskeletal architecture of glomerular podocytes and attenuate the transition from slit diaphragms to tight junctions; a key morphological change associated with proteinuria [96]. Additionally, activation of the Nrf2/heme oxygenase‐1 (HO‐1) pathway suppresses ferroptosis in PTECs by reducing iron overload, increasing GSH, and regulating ferroptosis mediators, ultimately alleviating diabetic kidney injury, as shown by attenuated glomerular hypertrophy, mesangial matrix expansion, podocyte foot process effacement, and glomerular basement membrane thickening [97]. These findings indicate that the differential roles of Nrf2 in regulating ferroptosis in the glomerulus and renal tubules may be related to disease stage and microenvironmental factors.

Notably, the core regulatory factors of ferroptosis are closely associated with the progression of DKD, highlighting their potential value as biomarkers. Studies have shown that elevated ACSL4 expression is positively correlated with increased proteinuria and negatively associated with serum albumin levels and estimated glomerular filtration rate, indicating a close link with accelerated renal function decline [98]. Meanwhile, GPX4, a key inhibitor of ferroptosis, is significantly downregulated in patients with DKD, and its expression level is closely correlated with disease progression [99].

### 4.3 Ferroptosis and DR

DR constitutes a significant microvascular complication of DM and is the primary cause of vision impairment, highlighting the necessity for prompt intervention [100]. Recent evidence has linked ferroptosis to DR pathogenesis, shedding light on underlying mechanisms and potential treatments [101, 102].

HG‐induced retinal oxidative stress causes DR [103]. A sustained oxidative milieu is created by chronic HG, which worsens mitochondrial membrane LPO and increases ROS via RPE cells [104]. Iron overload resulting from HG‐induced TFR1 upregulation and FTH1 degradation, as well as the Fenton reaction, promotes ferroptosis through lipid membrane oxidation [105]. Besides, NADPH oxidases 2 (NOX2) activation in HG causes ROS‐driven mitochondrial dysfunction, suppressing GPX4 and accelerating LPO/iron accumulation [106]. The high PUFA content in retinal lipids increases susceptibility to ferroptosis [107]. Accordingly, ferroptosis is widely thought to contribute to HG‐induced retinal neuron death, necessitating further research. In diabetic patients, elevated GMFB levels in vitreous fluid cause lysosomal alkalinization, inhibiting ACSL4 autophagy and leading to RPE ferroptosis [58]. By ubiquitinating GPX4, tripartite motif containing 46 (TRIM46) promotes human retinal capillary endothelial cells (HRCEC) ferroptosis and suppresses proliferation, suggesting that TRIM46/GPX4 is a potential DR therapeutic target [108]. BMS309403, a fatty acid–binding protein 4 (FABP4) inhibitor, reduces DR oxidative stress and LPO by inhibiting ferroptosis via proliferator‐activated receptor γ (PPARγ) activation [109]. Moreover, Fer‐1 activates the System Xc‐GPX4 antioxidant axis to reduce RPE damage [105]. Besides, it has been reported that Astragaloside‐IV suppresses Nrf2 to boost antioxidant defences and avert HG‐induced RPE ferroptosis [100]. Moreover, in the pathological process of DR, ferroptosis is also closely associated with the infiltration of immune cells. Studies indicate that the upregulation of heme oxygenase 1 (HMOX1) correlates with the infiltration of M2 macrophages and ferroptosis, which may play a pivotal role in the pathogenesis of proliferative DR [110].

In addition, the levels of ferroptosis‐related biomarkers, such as iron, LPO, and ROS, were significantly increased in the serum of patients with DR, while the level of GPX4 was decreased [111]. The changes in these biomarkers not only revealed the role of ferroptosis in DR but also provided a basis for its use as a diagnostic marker.

Research suggests that ROS‐mediated endothelial damage may induce ferroptosis, pyroptosis, and necroptosis, making the significance of ferroptosis in DR unclear [112]. Given that, these pathways may work alone or in combination, more research is warranted. Building upon current understanding that ferroptosis is regulated by LPO, oxidative stress, and molecular networks, elucidating its mechanisms may facilitate the advancement of DR treatments.

### 4.4 Ferroptosis and DCM

The diagnosis of DCM requires the exclusion of other diabetes‐related ventricular dysfunction conditions [113]. Its prevalence has reportedly increased alongside obesity and T2DM [114], elevating cardiovascular mortality in these patient populations.

Excessive iron accumulation can disrupt the antioxidant system in DCM, activate and exacerbate pathological processes such as excessive autophagy and mitochondrial dysfunction, triggering a cascade reaction that accelerates damage to the myocardium and microvasculature [115]. Chen et al. demonstrated that nicorandil blocks mitochondrial ferroptosis by promoting AMPKα1 phosphorylation, activating Pink1/Parkin‐mediated mitochondrial autophagy, and inhibiting ACSL4 transport. Thus, this drug improves cardiac microvascular function in DCM through the AMPKα1‐Parkin‐ACSL4 signalling pathway [116]. Additional studies indicate that lysine acetyltransferase 2A (Kat2a) enhances histone acetylation modifications through m6A modification, such as H3K27ac and H3K9ac, thereby promoting the transcription of TFR and HMOX1, ultimately triggering ferroptosis. This reveals that epigenetic regulation plays a role in the progression of DCM [117].

In diabetic animal models, abnormal ROS accumulation in myocardial tissues increases oxidative stress markers such as carbonylated proteins and fibrosis markers, including collagen type III, triggering the ferroptosis cascade [118]. The iron chelator deferiprone reduces fibrosis markers like collagen IV and monocyte chemoattractant protein‐1 (MCP1) and suppresses NF‐κB/Cyclooxygenase‐2 (COX‐2) activity, indicating potential for treating ferroptosis and inflammation [119]. Moreover, ferroptosis in diabetic hearts is induced by mitochondrial dysfunction, including mitochondrial loss, a decline in membrane potential, structural abnormalities, and excessive ROS production [120]. Sympathoadrenal overactivation, which causes abnormal catecholamine and growth hormone output, disrupts myocardial iron metabolism and ROS formation, indirectly affecting ferroptosis [121]. These findings collectively reveal the complex relationship between ferroptosis and neurohumoral dysfunction in DCM.

### 4.5 Ferroptosis and Diabetic Peripheral Neuropathy (DPN)

DPN is a prevalent chronic complication of diabetes, identified following the elimination of alternative causes [122].

According to recent research, ferroptosis, which includes metabolic dysregulation, oxidative stress, and aberrant calcium signalling, is a key factor in DPN pathophysiology. Tang et al. found that reduced Nrf2/GPX4 expression and ROS accumulation correlated with elevated P2Y purinergic receptor 14 (P2Y14) receptors and IL‐1β in the satellite glial cells of the dorsal root ganglia in diabetic rats [123], suggesting the Nrf2/GPX4 pathway is implicated in DPN. The Kelch‐like ECH‐associated protein 1 (Keap1)/Nrf2‐activating polysaccharide from *Lithospermum erythrorhizon* further supports this protective mechanism [124]. Lin et al. indicated that endoneurial hypoxia increases ROS and iron metabolism‐related proteins such as ferritin heavy chain (FHC), ferritin light chain (FLC), and TFR. while reducing GPX4, GSH, and SLC7A11 levels. These changes cause mitochondrial malfunction and reduced antioxidant capacity in cells, subsequently leading to ferroptosis in cerebral cortical neurons [125]. These findings indicate that the dysregulation of iron metabolism and ferroptosis may significantly contribute to the aetiology and progression of DPN, as diabetes can lead to persistent hypoxia in the endoneurium.

Furthermore, excessive polyol pathway activation depletes NADPH, thereby inhibiting GSH synthesis [126], while myo‐inositol deficiency epigenetically silences SLC7A11, synergizing with ACSL4‐dependent LPO [127]. Ferroptosis is thus strongly correlated with metabolic dysregulation. Calcium overload in dorsal root ganglion (DRG) neurons and increased dynamin‐related protein 1 (Drp1) phosphorylation drive small‐fibre degeneration. Targeting the mitochondrial calcium uniporter improves mechanical allodynia, indicating that calcium homeostasis is a critical node for blocking the ferroptosis‐neuropathy axis [128].

### 4.6 Ferroptosis and Diabetic Encephalopathy (DE)

Ferroptosis serves as a bidirectional regulator in the pathophysiology of diabetic cerebrovascular disease. Hepatic hepcidin, upregulated by insulin resistance, inhibits FPN1, causing brain iron overload [129]. Excess iron generates hydroxyl radicals via Fenton reactions, damaging the blood–brain barrier (BBB) and vascular endothelial tight junctions [130]. Concurrently, reduced GSH/GSSG ratios exacerbate neuronal redox imbalance, thereby worsening cerebrovascular pathology [131]. Furthermore, Hong et al. found that when the NOD‐like receptor family pyrin domain‐containing 3 (NLRP3) inflammasome in microglia is activated by HG, IL‐1β is released. This suppresses the system Xc/GSH antioxidant system and works in tandem with iron overload to hasten ferroptosis. Due to glucose metabolic inefficiency, patients with diabetes and stroke typically display a vicious iron‐inflammation cycle in the brain. Ferroptosis networks are modulated, and ischemic stroke outcomes are improved by interventions that target NLRP3 [132], indicating that ferroptosis serves a dual function in DCD as both a therapeutic target and a pathogenic driver.

Clinical and experimental studies have demonstrated that hippocampal iron overload drives DACD via upregulated ferroptosis markers Prostaglandin‐endoperoxide synthase 2 (PTGS2)/ACSL4 [54]. By breaking down synaptic proteins and causing dendritic spine loss, ferroptosis mechanistically causes deficiencies in spatial memory. A cognitive improvement rate of more than 35% is attained by AMPK agonists that activate GPX4 to inhibit LPO [133], demonstrating the therapeutic modifiability of the ferroptosis pathway.

### 4.7 Ferroptosis and Other Diabetic Complications

Diabetic foot (DF) is the term used to describe infections, ulcers, or tissue destruction in the distal foot and ankle areas brought on by peripheral vascular disease and/or diabetes‐associated neuropathy. In extreme situations, DF can lead to amputation [134]. By regulating the disruption of wound microenvironment homeostasis, ferroptosis has been shown in recent research to have a crucial regulatory function in the delayed healing of DF wounds.

HG‐induced iron overload exacerbates LPO and creates a ferroptosis‐driven microenvironment with ROS [135]. Exosomal circular RNAs derived from bone marrow stromal cells suppress ferroptosis via the Nrf2 pathway, thereby enhancing wound healing via angiogenesis [136]. Li et al. discovered that HG induces ferroptosis in fibroblasts and endothelial cells, marked by the overexpression of inflammatory cytokines and MDA, alongside the downregulation of GPX4 and SLC7A11. The upregulation of Fer‐1 activates the phosphatidylinositol 3‐kinase (PI3K)/AKT pathway and polarises M2 macrophages, thereby expediting wound healing [137]. Besides, vitamin D deficiency increases infection risk, and immune dysfunction disrupts the epidermal barrier [138]. The ferroptosis cascade is exacerbated by inflammatory mediators and cell activation [139]. Autophagy‐dependent ferroptosis via the sirtuin 1 (SIRT1)/Nrf2/p62 pathway promotes wound repair [140], suggesting ferroptosis regulation has reciprocal effects. Ferroptosis may delay diabetic ulcer healing and be a treatment target for diabetes.

The pathophysiology of diabetic osteoporosis (DOP) is a complex process. Based on the current understanding that diabetes worsens iron overload [141], Jiang et al. developed a mouse model of iron overload using dextran iron. Excess iron was observed to trigger osteoblast ferroptosis via the production of ROS bursts, MDA accumulation, and alterations in ferroptosis markers such as ACSL4 and GPX4. Therefore, osteoblast development and mineralisation were suppressed, and changes in mitochondrial ultrastructure were observed. As expected, DFO and Fer‐1 reversed these effects [44]. Therefore, osteoblast ferroptosis is crucial in the development of iron overload‐induced osteoporosis in diabetics. Preserving iron homeostasis and addressing osteoblast ferroptosis may prevent or treat iron overload‐induced osteoporosis in diabetes.

In summary, targeted regulation of ferroptosis‐related pathways may represent a potential therapeutic strategy for improving diabetes and its complications. However, current evidence supporting the modulation of ferroptosis primarily originates from in vitro studies or animal models, with no clinical research data available to date. Consequently, further mechanistic exploration and clinical validation are required for ferroptosis‐related targets suitable for clinical intervention.

## 5 Drugs Against Ferroptosis in DM and Its Complications

**FIGURE 4:**
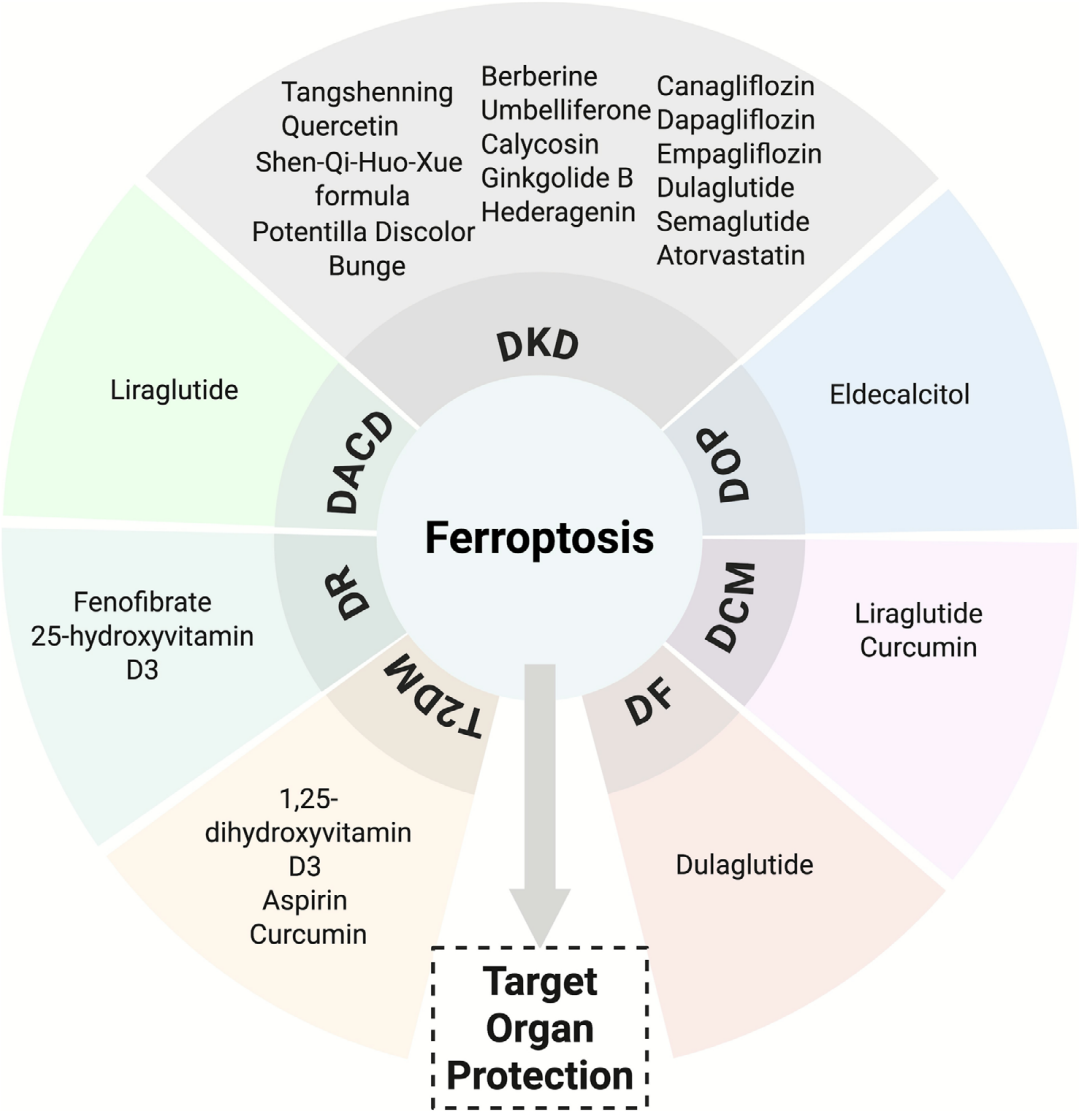
Drugs implicated in ferroptosis regulation in diabetes and its complications. Drugs targeting ferroptosis regulatory pathways have applications in diabetes and its complications, including diabetic retinopathy (DR), diabetes‐associated cognitive dysfunction (DACD), diabetic kidney disease (DKD), diabetic osteoporosis (DOP), diabetic cardiomyopathy (DCM), and diabetic foot (DF), including both traditional Chinese medicine (TCM) and Western pharmaceuticals.

Pharmacological interventions targeting ferroptosis for the management of DM and its complications are Western medicine and TCM. These medications have demonstrated significant enhancements in metabolic dysfunction and renal damage in both in vitro and in vivo environments. To mitigate the advancement of DM and its ramifications, ferroptosis has surfaced as a prospective therapeutic target (Figure 4).

### 5.1 Western Medicine

In recent years, the regulatory mechanisms of ferroptosis have become a crucial focus in developing therapeutic strategies for DM and its complications. Diverse pharmacological strategies have demonstrated novel therapeutic potential for organ protection within conventional Western medicine by multitargeted modulation of ferroptosis‐related pathways (Table 1).

**TABLE 1:** Western medicine associated with DM and its complications by targeting ferroptosis.

Drugs	Diseases	Mechanisms	Experimental models	References
Canagliflozin	DKD	Improved FAO and via the FOXA1‐CPT1A axis	db/db diabetic mice; HG‐stimulated renal HK‐2 cells	[142]
Canagliflozin	DCM	Balanced cardiac iron homeostasis and promoted the System Xc‐/GSH/GPX4 axis	STZ‐induced mice; HG‐stimulated H9C2 cardiomyocytes	[143]
Dapagliflozin	DKD	Downregulation of the HIF1α/HO‐1 axis	STZ‐induced diabetic mice; HG and hyperlipidemic‐treated HK‐2 cells	[144]
Dapagliflozin	DCM	Elevation of GPX4 and suppression of ACSL4 levels	Adult diabetic patients with HFrEF	[145]
Empagliflozin	DKD	Promoted the AMPK‐mediated Nrf2 activation pathway	STZ‐induced diabetic mice; HG‐stimulated renal HK‐2 cells	[146]
Empagliflozin	DCM	Elevated GPX4 and decreased ACSL4 levels	Adult diabetic patients with HFrEF	[145]
Dulaglutide	DF	Activated the Nrf2/GPX4/SLC7A11 pathway	db/db diabetic mice; HG‐induced keratinocytes	[147]
Liraglutide	DCM	Increase in the protein levels of Cyto‐Nrf2, Nu‐Nrf2, PTGS2, FTH‐1, GPX4 and reduced LPO	Spontaneous diabetes Goto‐Kakizaki (GK) rats HG‐stimulated H9C2 cardiomyocytes	[148]
Liraglutide	DACD	Decreased levels of TFR1, ACSL4 and upregulated FPN1, FTH, GPX4, SLC7A11	db/db diabetic mice	[149]
Semaglutide	DKD	Activated KLB/AMPK/Nrf2 signalling pathway	STZ‐induced diabetic mice, HG‐treated HK‐2 cells	[150]
1,25‐dihydroxyvitamin D3(1,25D)	T2DM	Increased in GPX4 expression; Decreased in ROS, iron and ACSL4 expression	STZ‐induced diabetic rat; pancreatic β cells	[151]
Eldecalcitol	DOP	Increased GPX4 levels and inhibited	STZ‐induced diabetic rat; HG‐induced rat primary osteoblasts	[152]
25‐hydroxyvitamin D3	DR	Reduced Fe^2+^ level and increased GPX4 and SLC7A11 expression	HG‐induced human retinal microvascular endothelial cells (hRMVECs)	[153]
Fenofibrate	DKD	Increased Nrf2	STZ‐induced DBA/2J diabetic mice; HG‐induced HK‐2 cells	[154]
Fenofibrate	DR	Reduced ROS production and NADPH oxidase activity, alleviated oxidative stress	STZ‐induced diabetic rat; HG‐induced ARPE‐19 cells	[155]
Atorvastatin	DKD	Reduced renal ROS, iron accumulation and MDA,4‐HNE, TFR1, and increased GPX4, NRF2, and FTH	STZ‐induced diabetic mice; HG‐stimulated renal HK‐2 cells	[156]
Aspirin	T2DM	Inhibited COX2 activation and enhanced GPX4 and SLC7A11 expression	STZ‐induced DBA/2J diabetic mice; HG‐induced HK‐2 cells	[157]
Melatonin	DOP	Activated the Nrf2/HO‐1 pathway	Sprague Dawley rats; The osteoblastic cell line MC3T3‐E1	[158]

Multifaceted regulation of ferroptosis is illustrated in traditional Western medicine by sodium‐glucose cotransporter‐2 inhibitors (SGLT2i). Canagliflozin mitigates ferroptosis in RTECs in individuals with DKD by activating the Forkhead box A1 (FOXA1)/carnitine palmitoyltransferase 1A (CPT1A) pathway to promote fatty acid oxidation (FAO) [142]. Furthermore, by modulating iron homeostasis and activating the Xc‐/GSH/GPX4 antioxidant pathway, Cana diminishes myocardial ferroptosis and offers cardiovascular protection in a DCM model [143]. DAPA and empagliflozin inhibit LPO and iron overload via the Hypoxia‐inducible factor‐1 alpha (HIF1α)/HO‐1 and AMPK/Nrf2 pathways, respectively. This process is achieved by upregulating GPX4 expression and inhibiting ACSL4, thereby diminishing ferroptosis and ameliorating tissue damage in DKD and DCM [144]. The modulation of ferroptosis illustrates the therapeutic potential of SGLT2 inhibitors in organ protection.

Various glucagon‐like peptide‐1 (GLP‐1) receptor agonist delivery methods regulate ferroptosis, with organ‐protective benefits in diabetes and its complications. Dulaglutide stimulates the Nrf2/GPX4/SLC7A11 pathway, which suppresses Nrf2‐dependent ferroptosis in HG, thereby accelerating diabetic wound healing [145]. Liraglutide (LIRA) stimulates the Nrf2 signalling pathway and increases the expression of antiferroptotic proteins such as Cyto‐NRF2, Nu‐Nrf2, PTGS2, FTH‐1, and GPX4, thereby diminishing ROS and LPO in DCM. This increases cardiac dysfunction, hinders remodelling, and lowers ferroptosis [146]. LIRA diminishes ROS and MDA while enhancing serum and hippocampal SOD and GSH‐Px activity, thereby reducing iron overload in DACD. Downregulating TFR1, upregulating FPN1 and FTH, and improving mitochondrial iron management alter iron metabolism and reduce cognitive deficits in diabetic mice [147]. By phosphorylating cAMP response element‐binding protein (CREB) and protein kinase A (PKA), semaglutide alters iron metabolism, fatty acid synthesis, and antioxidant responses. This increases klotho (KLB) expression and AMPK signalling. In DKD, blocking ferroptosis was observed to diminish renal inflammation and fibrosis [148]. The above studies overlap in their assertion that GLP‐1 receptor agonists modulate ferroptosis, thereby reducing multiorgan damage in diabetes.

Recent studies indicate that 1,25‐dihydroxyvitamin D3 [1,25(OH)_2_D_3_] may mitigate ferroptosis in pancreatic β cells of T2DM patients by enhancing GPX4 expression and reducing levels of ROS, iron, and ACSL4 by downregulating FoxO transcription factor 1 (FOXO1) [149]. Eldecalcitol, a vitamin D analogue, activates the HIF1α pathway, increasing GPX4 levels and preventing osteoblast ferroptosis [150]. In DR, 25‐hydroxyvitamin D3 downregulates miR‐93, thereby preventing ferroptosis and oxidative stress in retinal microvascular endothelial cells. Comprehensive analysis revealed increased GSH, GPX4, and SLC7A11 expression, and lower ROS, MDA, and Fe^2+^ levels [151]. Fenofibrate reportedly inhibits ferroptosis in DKD and DR by activating the Nrf2 pathway to combat oxidative stress, LPO, and iron excess, slowing disease progression [152, 153]. Moreover, aspirin delays DKD and prevents ferroptosis in renal tubular HK‐2 cells under hyperglycemic conditions by inhibiting COX‐2 [154]. Similarly, atorvastatin alleviates DKD by reducing renal ROS, iron buildup, LPO products such as MDA and 4‐hydroxynonenal (4‐HNE), along with elevated levels of antioxidant proteins like GPX4 and Nrf2, and proteins involved in iron metabolism such as FTH. It also reduces mitochondrial damage, providing a mechanistic approach to DKD prevention and treatment [155].

### 5.2 TCM Components

Recent research has demonstrated that TCM active constituents modulate ferroptosis through multiple targets and pathways, offering new potential therapeutic options for diabetes and its complications (Table 2). In diabetic models, curcumin reduces HG‐induced LPO and GPX4 inactivation by inhibiting ferroptosis and promoting Nrf2 nuclear translocation. This protects pancreatic function and reduces DCM and myocardial injury [156, 157]. In this context, Berberine (BBR) and umbelliferone reportedly improve renal function in DKD by upregulating Nrf2, HO‐1, and GPX4 and downregulating ACSL4, reducing oxidative stress, iron accumulation, and tubular injury [158, 159]. Moreover, calycosin prevents DKD by preventing HG‐induced GPX4 and MDA decreases and lipid ROS accumulation [160]. Ginkgolide B (GB) in DKD attenuates tubular ferroptosis and fibrosis by enhancing the expression of iron metabolism‐related proteins, including GPX4 and FTH1, reducing fibrotic markers like collagen α1 and α‐SMA, and lowering TFR1 levels [161]. Hederagenin (HDG) prevents renal fibrosis caused by oxidative stress [162]. Potentilla Discolour Bunge (PDB) reduces MDA, Fe^2+^, and ROS in renal tissues and restores antioxidant systems like Nrf2, HO‐1, GPX4, and SLC7A11 [163]. Tangshenning (TSN) has been shown to mitigate kidney damage in DKD by restoring mitochondrial function and inhibiting ferroptosis in PTECs through the Sestrin2/AMPK/PGC‐1α pathway [164]. A separate study demonstrated the Shen‐Qi‐Huo‐Xue formula (SQHXF) reduces tubular ferroptosis, proteinuria, and EMT in DKD by adjusting the HIF‐1α/HIF‐2α equilibrium [165]. Besides, melatonin inhibits retinal cell ferroptosis in DR by activating Nrf2/HO‐1 [166]. Through the AMPK/Nrf2 pathway, sulforaphane reduces cardiomyocyte ferroptosis and heart failure in dilated cardiomyopathy. In DKD, quercetin (QCT) prevents ferroptosis through the downregulation of TFR1 and upregulating GPX4, FTH1, and SLC7A11, indicating novel protective mechanisms [163, 167, 168].

**TABLE 2:** TCM associated with DM and its complications by targeting ferroptosis.

Drugs	Diseases	Mechanisms	Experimental models	References
Curcumin	T2DM	Prevented GSH depletion, GPX4 inactivation, and LPO	MIN6 cells STZ‐induced diabetic mice INS‐1 cells	[159]
Curcumin	DCM	Promoted Nrf2/HO‐1 pathway and upregulated GPX4 levels	DCM rabbits HG‐stimulated H9C2 cardiomyocytes	[160]
Berberine	DKD	Increased in Nrf2, HO‐1 and Gpx4 expression, and decreased in ACSL4 expression	STZ‐induced DKD rat model HG‐treated HK‐2 cells	[161]
Umbelliferone	DKD	Activated of the Nrf‐2/HO‐1 pathway	db/db diabetic mice HG‐stimulated HK‐2 cells	[162]
Calycosin	DKD	Increased in GPX4, MDA and decreased in lipid ROS	db/db diabetic mice HG‐stimulated renal HK‐2 cells	[163]
Ginkgolide B	DKD	Inhibited the ubiquitination of GPX4	db/db diabetic mice HG‐stimulated MPC5 cells	[164]
Hederagenin	DKD	Activated the Smad3/Nox4/SLC7A11 pathway	STZ‐induced diabetic mice HG‐treated HK‐2 cells	[165]
Potentilla Discolour Bunge	DKD	Upregulated the Nrf2 signalling pathway and inhibited ferroptosis initiation	STZ‐induced DKD rat model HG‐treated HK‐2 cells	[166]
Tangshenning	DKD	Down‐regulation of SLC7A11 and GPX	KK‐Ay mice Hyperglycemic‐treated HK‐2 cells	[167]
Quercetin	DKD	Regulated the Nrf2/HO‐1 pathway	db/db diabetic mice HG‐treated HK‐2 cells	[168]
Shen‐Qi‐Huo‐Xue formula	DKD	Increased GPX4 expression and GSH content, decreased the levels of ROS, Fe^2+^, MDA, and LPO	KK‐Ay mice (A spontaneous DKD mouse model)	[169]
Sulforaphane	DCM	Upregulated ferritin and SLC7A11 levels and activated AMPK/Nrf2 pathway	DCM mice AGE‐treated ECTs	[170]

These findings demonstrate that TCM components modulate ferroptosis via antioxidant defences, lipid homeostasis, iron metabolism, and key signalling pathways, establishing a robust theoretical foundation and practical targets for multitargeted interventions in diabetes and its complications. In short, drugs or formulations targeting ferroptosis may offer new therapeutic avenues for diabetes patients.

## 6 Conclusions and Future Perspectives

This review outlines the primary molecular mechanisms of ferroptosis, including anomalies in the amino acid antioxidant system, disrupted iron metabolism, and the accumulation of lipid peroxides. This review examines these pathways to elucidate their role in the pathogenesis of DM and its complications while assessing the potential of novel targeted interventions for clinical translation. The relationships between ferroptosis and emerging fields of research, including MAMs, ERS, MLIs, and LD‐MIs, are first explored. These findings may inform the development of novel therapeutic strategies aimed at the pathways involved in ferroptosis in diabetes and related disorders.

The role of ferroptosis in diabetes and its related consequences has attracted significant interest; however, numerous challenges remain regarding mechanistic clarification and clinical application in ongoing research. Nutrition, chronic inflammation, and genetic predisposition collectively regulate iron metabolism homeostasis in individuals with diabetes. Nevertheless, most existing research has relied on models such as *db/db* diabetic mice, STZ‐induced diabetes, or HG‐treated in vitro systems. The spatiotemporal variation of iron metabolism observed in human disease cannot be entirely replicated by these experimental models, thereby significantly limiting the applicability of findings from animal models in clinical contexts. Secondly, while therapeutic interventions such as insulin and SGLT2i may enhance metabolic dysfunction and thereby indirectly mitigate oxidative stress, their molecular targets remain inadequately linked to critical ferroptosis pathways, including GPX4‐dependent antioxidant systems and ACSL4‐mediated PUFA overload. The absence of a definitive molecular connection precludes the ability to draw conclusive insights about whether the regulation of ferroptosis underpins the efficacy of existing treatments. These findings also limit the clinical application of direct ferroptosis inhibitors, such as Fer‐1, which require validation for stability, tissue selectivity, and long‐term safety for human use, despite their demonstrated potential in preclinical models.

To overcome the challenges in ferroptosis research and its therapeutic applications, the development of ferroptosis‐targeting drugs with optimised pharmacokinetic properties is essential. Nanoparticle encapsulation can enhance drug distribution and bioavailability, potentially improving therapeutic outcomes [169]. However, challenges persist, including the need for more precise targeting to ensure selective delivery and reduce toxicity. Pharmacokinetic limitations of some inducers, like curcumin's poor solubility and bioavailability, still hinder clinical efficacy [170]. Moreover, ferroptosis involves complex signalling pathways, such as the system Xc‐/GSH/GPX4 axis and AMPK pathway, which can interact broadly with cellular processes [171], increasing the risk of off‐target effects and toxicity. Thus, developing ferroptosis‐targeting drugs requires not only improving pharmacokinetics but also minimising off‐target interactions and ensuring clinical safety.

In summary, future research must concentrate on the following areas to address these challenges: examining the potential multipathway effects of ferroptosis inhibitors in alleviating metabolic dysfunction through their combination with existing antidiabetic drugs, such as SGLT2i. Furthermore, integrating patient genetic polymorphisms, such as variations in the *GPX4* gene, with complication risk assessment could facilitate the development of tailored intervention strategies for diverse complications. For example, prioritising kidney‐protective ferroptosis inhibitors that focus on renal iron homeostasis or the LPO pathway may be beneficial for individuals at elevated risk of developing diabetic nephropathy due to ferroptosis. Altering iron homeostasis in cardiomyocytes may emerge as a primary strategy for addressing cardiovascular issues to mitigate damage induced by ferroptosis. These precise medical techniques warrant additional comprehensive examination.

## Author Contributions

Zheng Wang, Qi Feng, and Guolan Xing initiated the study design and oversaw the overall research direction. Zheng Wang, Ying Zhang, Yue Xu, Mengyuan Yang, Yuan Wei, Yuan Li, Tongyue Yang, Xu Wang, and Chaona Yang contributed to the drafting of specific manuscript sections. Qi Feng and Guolan Xing, as corresponding authors, provided strategic guidance, conducted rigorous quality control, and finalised the intellectual content. All authors participated in critical discussions and approved the final version of the manuscript prior to submission.

## Funding

This work was supported by the National Natural Science Foundation General Program (No. 82270766), the Scientific and Technological Innovation Young Top Talents in Central Plains, the Young and Middle‐Aged Innovation Talents of Health Science and Technology Project in Henan Province (No. YQRC2024011), the “Three 100” Plan Overseas Study Program for Medical Science and Technology Talents of Henan Province Medical Sciences Academy (H20250220), and the Excellent Young Scientists Fund Program of the Natural Science Foundation of Henan Province (No. 252300421112).

## Conflicts of Interest

The authors declare no conflicts of interest.

## Data Availability

Data sharing not applicable to this article as no datasets were generated or analysed during the current study.
